# Current Approaches to the Management of Acute Surgical and Medical Emergencies: A Structured Narrative Review

**DOI:** 10.7759/cureus.107799

**Published:** 2026-04-27

**Authors:** Sanjit Prasad, Pooja Verma, Fidvi Fazal, Parv Rasiklal Raiyani, Marripati Sneha Priya, Tushar Harivadan Shah

**Affiliations:** 1 Department of General Surgery, All India Institute of Medical Sciences, Patna, Patna, IND; 2 Department of Emergency Medicine, Santosh Medical College, Ghaziabad, IND; 3 Department of General Surgery, Government Medical College, Kannur, Kannur, IND; 4 Department of Critical Care Medicine, MGM New Bombay Hospital, Navi Mumbai, IND; 5 Department of Anaesthesiology and Critical Care, Command Hospital, Atal Bihari Vajpaye Medical University, Lucknow, IND; 6 Department of General Medicine, Dr. ND Desai Faculty of Medical Science and Research, Dharmsinh Desai University, Nadiad, IND

**Keywords:** acute care, emergency medicine, multidisciplinary management, risk stratification, surgical emergencies

## Abstract

Acute surgical and medical emergencies constitute a major cause of morbidity and mortality, placing demands on healthcare systems due to sudden onset, rapid progression, and the requirement for immediate intervention. Optimal outcomes depend on early recognition, structured assessment, and coordinated multidisciplinary care involving emergency medicine, surgical teams, and critical care services. This structured narrative review provides an evidence-informed overview of contemporary approaches to the assessment, diagnosis, and management of acute surgical and medical emergencies, with emphasis on clinically applicable pathways. The review addresses key domains including epidemiology and risk stratification, principles of initial assessment and stabilisation, and diagnostic strategies incorporating laboratory biomarkers and advanced imaging modalities. Management of common acute surgical and medical emergencies is examined, alongside current pharmacological interventions such as antimicrobial therapy, analgesia, sedation, and anticoagulation within high-acuity settings. The role of interdisciplinary integration, post-intervention monitoring, and timely escalation of care is highlighted as a determinant of continuity and outcome optimisation. Emerging innovations, including artificial intelligence-based clinical decision support systems, tele-emergency medicine, and minimally invasive emergency procedures, are discussed for their potential to enhance diagnostic accuracy, triage efficiency, and therapeutic precision. Persistent challenges include heterogeneous presentations, inconsistent guideline implementation, and unequal access to emergency services. Future efforts should prioritize standardized pathways, validated technologies, and integrated care models.

## Introduction and background

Acute surgical and medical emergencies represent one of the most critical groups of clinical conditions characterised by rapid onset, rapid physiological decline, and a high probability of morbidity and mortality in the absence of timely intervention [1]. This review focuses on high-acuity adult emergencies encountered in emergency and critical care settings, including acute abdominal surgical conditions, major trauma, cardiovascular and thromboembolic events, neurological crises, and severe infections such as sepsis, while elective, chronic, and highly specialised subsystems are not addressed in detail. Such emergencies include traumatic injuries, acute disorders of the abdomen, cardiovascular conditions, neurological crises, severe infections, and multisystem dysfunctions that require prompt clinical evaluation and decisive management [2]. The main common feature of these conditions is their extreme time dependence, where delays in diagnosis or treatment are associated with worse outcomes, prolonged hospitalisation, and increased mortality rates [3]. Consequently, modern emergency care is structured around protocol-driven approaches, rapid clinical decision-making, and multidisciplinary interventions aimed at stabilisation and prevention of irreversible harm.

The number of acute surgical and medical emergencies worldwide continues to increase due to population ageing, urbanisation, changing lifestyles, and the rising prevalence of chronic non-communicable diseases [4]. A significant proportion of emergency department admissions and hospital-related mortality is attributed to cardiovascular emergencies, trauma, sepsis, and acute neurological conditions [5]. Middle- and low-income countries face challenges related to limited access to emergency surgical care, delayed presentation, and insufficient intensive care infrastructure [6]. In contrast, high-income healthcare systems encounter persistent issues such as emergency department overcrowding, resource constraints, and increasingly complex patient profiles [7]. These disparities highlight the need for adaptable and context-sensitive emergency care systems.

Early identification and prompt response remain key determinants of survival and functional recovery in emergency settings [8]. Structured assessment frameworks, including airway, breathing, circulation, neurological status, and exposure, provide a systematic basis for prioritisation and timely intervention [9]. Advances in point-of-care testing, diagnostic imaging, and biomarker-based risk assessment have improved diagnostic accuracy and facilitated earlier initiation of targeted therapies [10]. Concurrent developments in damage control surgery, trauma resuscitation, and protocol-based critical care have contributed to measurable reductions in mortality among high-risk emergency populations.

Despite these advances, emergency care delivery remains challenged by clinical heterogeneity, overlapping symptomatology, and evolving disease patterns [11,12]. Emerging technologies, including artificial intelligence-supported decision systems, digital twin modelling, and advanced monitoring protocols, are being explored as potential tools to enhance prediction, triage accuracy, and personalised care in emergency settings [13]. However, their integration into routine clinical practice is limited by infrastructure constraints, training requirements, and variability in real-world validation [14]. These factors add complexity to rapid decision-making in high-acuity environments.

Certain areas continue to evolve within emergency care. Interactive wound dressings support early tissue stabilisation, reduce infection risk, and improve wound outcomes following trauma or surgery [15]. Acute cardiac syndromes such as Takotsubo syndrome may mimic myocardial infarction, requiring heightened clinical awareness and appropriate haemodynamic management [16]. The increasing use of artificial intelligence raises considerations related to accountability, transparency, and patient autonomy, particularly in time-critical settings [17]. Advances in cardiopulmonary resuscitation emphasise physiological optimisation, post-resuscitation care, and coordinated system-level responses to improve survival after cardiac arrest [18]. Established trauma frameworks, such as Advanced Trauma Life Support, remain central to structured initial assessment and prioritised care across emergency presentations [19,20]. Additionally, developments in biomaterials and 3D bioprinting represent emerging areas with potential future applications in acute tissue repair and regenerative strategies.

This review adopts a structured narrative approach, integrating surgical, medical, traumatic, and critical care emergencies through shared principles of assessment, stabilisation, and management, rather than addressing them as isolated specialties. Despite progress, gaps remain in integrated risk assessment across mixed surgical-medical emergencies, consistent implementation of evolving technologies, and equitable access to emergency care. Variability in guideline adherence, limited high-quality comparative evidence, and challenges in interdisciplinary coordination continue to affect outcome optimisation. Accordingly, this review aims to synthesise current practices, identify priority gaps, and highlight areas requiring further clinical and research focus.

Objectives of the review

This structured narrative review provides a clinically oriented overview of the available methods in evaluating and treating acute surgical and medical emergencies across major clinical areas. It is intended primarily as a clinically practical synthesis, focusing on integrating evidence-informed approaches to support real-world decision-making rather than performing formal comparative analysis across interventions. The review also points towards emerging trends and future priorities that can enhance the provision of emergency care in an evidence-based and integrated manner.

Methodology

The methodology applied in this review was a structured narrative approach with a defined process for literature identification, screening, selection, and thematic synthesis to examine current approaches in the management of medical and acute surgical emergencies. This approach was selected due to the considerable heterogeneity in clinical presentation, severity, patient populations, and care settings, which limits the feasibility of quantitative synthesis. The review focused on clinically relevant assessment frameworks, treatment pathways, and decision-making processes applicable to emergency practice. The methodology was designed to improve clarity and transparency by explicitly outlining the search, selection, and synthesis procedures.

The literature search was conducted using PubMed, Web of Science, and Scopus databases, covering publications from 2015 to 2025. The search strategy used combinations of keywords and Boolean operators, including (“acute medical emergencies” OR “surgical emergencies” OR “trauma management”) AND (“critical care” OR “resuscitation” OR “emergency management” OR “multidisciplinary care”). Filters were applied to include English-language, peer-reviewed human studies published between January 2015 and December 2025. The final search was completed in March 2026. Additional keywords were used to capture key domains such as diagnostic strategies, treatment pathways, and multidisciplinary care. Search terms were adjusted across databases to ensure appropriate coverage of relevant literature.

All identified records were screened initially by title and abstract, followed by full-text review for eligibility. Duplicate records were removed prior to screening. Study selection was conducted independently by two reviewers, with disagreements resolved through discussion and consensus. Predefined inclusion and exclusion criteria were applied consistently during screening to ensure selection transparency and reduce bias.

Eligible studies included clinical trials, observational studies, systematic and narrative reviews, consensus statements, and guideline-based publications relevant to emergency care practice. Non-clinical studies, case reports, preclinical research, and editorials were excluded. Study quality and level of evidence were considered during selection, with greater emphasis placed on higher-quality evidence such as clinical trials, systematic reviews, and guideline-based studies, followed by relevant observational research. Studies were prioritised based on methodological strength, clinical relevance, and consistency of findings across sources.

Evidence was synthesised using a thematic approach to allow structured comparison across different emergency conditions and care settings. Themes were developed based on recurring clinical concepts identified during full-text review, including epidemiology, risk stratification, initial assessment, diagnostic approaches, management strategies, and system-level integration. Thematic grouping was performed independently by two reviewers and refined through consensus to improve consistency and clarity. Consideration was given to differences in study design, healthcare settings, and reporting variability during interpretation. The synthesis focused on identifying common patterns across studies to support balanced and clinically applicable conclusions.

## Review

Epidemiology and risk stratification

Emergencies in acute surgery and acute medical care exhibit different incidence and mortality rates, which are dependent on underlying pathology, patient variables, and medical conditions [21]. Representative neurological emergencies encountered in acute settings include stroke, traumatic brain injury, and acute spinal cord compression, where delayed diagnosis and progressive neurological impairment can lead to severe functional deterioration and increased perioperative risk, particularly in elderly populations [22]. Cardiovascular and thromboembolic crises continue to be among the most frequent causes of mortality related to emergencies, with venous thromboembolism being a significant contributor to in-hospital death when risk factors are not promptly identified [23]. Access to rapid diagnostics, procedural intervention, and critical care support also affects mortality patterns.

Emergency risk profile is greatly influenced by demographic and clinical factors such as age, sex, socioeconomic status, and comorbid conditions [24]. Ageing populations are particularly vulnerable to degenerative, thrombotic, and cardiovascular emergencies, often in the presence of multimorbidity [25]. Comorbid conditions, including chronic kidney disease, further complicate clinical decision-making, particularly in diagnostic processes where contrast-enhanced imaging may pose additional risks and influence management strategies [14]. Cardiometabolic diseases, reduced mobility, malignancy, and inflammatory conditions also increase susceptibility to adverse outcomes in acute settings [7]. In parallel, emerging technologies, including advanced predictive models and artificial intelligence-based tools, are increasingly being explored to support risk stratification, particularly in high-risk cardiac populations. These technologies aim to enhance early identification of deterioration and inform clinical decision-making, although their integration into routine emergency practice remains variable.

The risk stratification of emergencies in relation to triage systems and severity scoring tools is still very central [12]. These frameworks guide clinicians to focus on the priorities of care, resource allocation, and predicting outcomes in a wide range of emergencies [8]. Physiological parameters, comorbidity burden, and situational factors, such as environmental and behavioural factors that influence patient response in an emergency, are growing in risk stratification [13]. The systematic triage frameworks and evidence-based risk assessment instruments help in the prompt detection of high-risk patients and enable prompt intervention and enhanced outcome maximisation in the emergency care systems. Key variables are summarised in Table 1.

**Table 1:** Risk Stratification Variables and Clinical Utility CKD: Chronic kidney disease; GCS: Glasgow Coma Scale; CT: Computed tomography; ICU: Intensive care unit

Risk Factor / Variable	Clinical Context	Associated Outcome / Utility	Reference
Age (≥65 years)	Multisystem emergencies, trauma, sepsis	Increased mortality, higher ICU admission rates	[26]
Comorbidities (e.g., CKD, cardiometabolic disease)	Acute medical and surgical emergencies	Increased complication risk, influences diagnostic and treatment decisions	[23]
Physiological parameters (e.g., hypotension, tachycardia, hypoxia)	Triage and early assessment	Predictors of clinical deterioration and need for urgent intervention	[18]
Neurological status (e.g., GCS score)	Trauma, stroke, CNS emergencies	Correlates with severity and prognosis	[7]
Biomarkers (e.g., lactate, inflammatory markers)	Sepsis, shock states	Early identification of high-risk patients and monitoring response to treatment	[22]
Imaging findings (CT, ultrasound)	Trauma, abdominal and neurological emergencies	Guides definitive diagnosis and intervention planning	[23]
Socioeconomic and access factors	Delayed presentation settings	Associated with late diagnosis and poorer outcomes	[13]

Initial assessment and stabilisation principles

Universally accepted initial assessment principles form the foundation of effective management in acute surgical and medical emergencies and are applied consistently across all emergency presentations, with prioritisation of airway, breathing, and circulation as the key component [26]. Structured trauma and emergency algorithms emphasise rapid identification of airway compromise, respiratory insufficiency, and circulatory instability to prevent secondary injury and early deterioration [27]. Immediate interventions such as airway support, oxygen administration, and ventilatory assistance are essential to restore physiological homeostasis, particularly in patients presenting with altered consciousness or cardiorespiratory collapse [28]. Early control of external bleeding and initial management of soft tissue injuries are also integral to primary stabilisation.

Following this universal initial assessment, condition-specific stabilisation strategies are implemented based on the underlying diagnosis and physiological derangement. Hemodynamic stabilisation aims to maintain adequate tissue perfusion and oxygen delivery, with tailored approaches including fluid resuscitation, vasopressor support, and targeted management of shock states [12,5]. In common high-acuity emergency conditions such as septic shock, major trauma, and acute coronary syndromes, careful haemodynamic monitoring and timely, protocol-driven interventions are essential to prevent organ dysfunction and improve survival outcomes. Advances in cardiopulmonary resuscitation further highlight the importance of optimised circulation, high-quality chest compressions, and structured post-resuscitation care [18]. Point-of-care diagnostics play a critical role in guiding both general and condition-specific interventions by enabling rapid bedside assessment of physiological status and supporting timely escalation of care [8]. The integration of advanced diagnostic and decision-support technologies continues to evolve, although clinical oversight remains essential in high-acuity emergency settings [17]. Emerging innovations, including biomaterial and regenerative approaches, may further expand the scope of early stabilisation strategies in the future.

Diagnostic approaches in emergency settings

Effective management of acute surgical and medical emergencies depends on accurate and timely diagnosis, as delays or diagnostic errors significantly worsen clinical outcomes [29].

Laboratory Diagnostics

Biomarkers play a central role in early risk identification and clinical decision-making by providing rapid insight into underlying physiological disturbances [30]. Their evaluation assists in differentiating inflammatory, metabolic, infectious, and neurological emergencies, thereby supporting prioritisation of urgent interventions [13]. Metabolic abnormalities and vitamin deficiencies presenting with acute neurological symptoms require prompt identification to prevent irreversible damage, reinforcing the importance of targeted laboratory testing in emergency settings [20].

Bedside Imaging

Point-of-care ultrasound serves as a rapid, bedside diagnostic tool for assessing hemodynamic status, abdominal pathology, and cardiac function, enabling immediate clinical decision-making in acute scenarios [9].

Cross-Sectional Imaging

Computed tomography remains the first-line imaging modality in trauma, acute neurological deficits, and abdominal emergencies due to its high diagnostic accuracy and rapid acquisition [24]. Magnetic resonance imaging provides superior soft tissue and neurological detail in selected time-sensitive conditions such as encephalopathy and spinal emergencies, although its use may be limited by availability and longer acquisition times [15].

Disease-Specific Diagnostic Algorithms

Time-critical diagnostic pathways integrating laboratory findings, imaging modalities, and clinical evaluation help streamline emergency workflows and improve triage efficiency [31]. These structured approaches enhance situational awareness, reduce cognitive burden, and facilitate rapid decision-making in high-pressure environments [32]. Emerging computational tools and machine learning models are increasingly being explored to support diagnostic precision, although their routine integration into emergency practice remains evolving [23].

The combination of laboratory diagnostics, bedside and cross-sectional imaging, and structured algorithms forms the basis of efficient and timely emergency evaluation. Table 2 summarises key diagnostic tools and their clinical applications in emergency decision-making.

**Table 2:** Modalities, Applications, and Clinical Impact of Diagnostic Approaches in Emergency Settings CT: Computed tomography, MRI: Magnetic resonance imaging, ML: machine learning

Diagnostic Component	Primary Method	Emergency Application	Clinical Impact	Reference
Laboratory biomarkers	Metabolic and biochemical markers	Early differentiation of inflammatory, metabolic, infectious, and neurological emergencies	Enables rapid risk stratification and timely intervention	[22]
Point-of-care ultrasound	Bedside ultrasonography	Hemodynamic assessment, abdominal pathology, cardiac evaluation	Supports immediate clinical decision-making	[4]
CT	Cross-sectional imaging	Trauma, acute neurological deficits, abdominal emergencies	Provides rapid and high diagnostic accuracy	[7]
MRI	Advanced soft-tissue imaging	Encephalopathy, spinal and neurological emergencies	Enhances diagnostic precision in selected cases	[27]
Diagnostic algorithms	Integrated clinical pathways and ML-assisted models	Time-critical triage and workflow optimization	Improves diagnostic efficiency under high-pressure settings	[30]

Management of acute surgical emergencies

Acute surgical emergencies need to be managed through prompt diagnosis, timely operative decision-making, and a multidisciplinary approach to prevent progression to organ failure and mortality [33]. Management strategies vary according to the underlying pathology, with distinct priorities across major emergency conditions. Acute abdominal pathologies such as gastrointestinal perforation require urgent resuscitation, early initiation of broad-spectrum antimicrobial therapy, and prompt surgical exploration to control contamination and prevent sepsis progression [34]. In cases of bowel obstruction, management focuses on fluid resuscitation, electrolyte correction, gastric decompression, and timely operative intervention when there is evidence of strangulation or ischemia.

Trauma and hemorrhagic shock remain significant causes of surgical mortality in emergency settings [10]. Successful management emphasises rapid haemorrhage control, restoration of circulating volume, and prevention of secondary injury. Major hemorrhage requires immediate bleeding control using surgical or interventional techniques, activation of massive transfusion protocols, and correction of coagulopathy. Abbreviated surgical procedures and staged definitive repair, as part of damage control surgery, are employed in physiologically unstable patients [25]. Innovations in trauma services and perioperative care have reinforced the importance of early intervention, structured resuscitation strategies, and coordinated postoperative management [16]. Evidence-based wound care practices further contribute to tissue stabilisation, infection control, and improved recovery outcomes in the acute surgical setting.

Surgical emergencies can be systematically grouped based on clinical domain and acuity to guide prioritised diagnostic and management strategies.

Vascular Emergencies

Conditions such as acute aortic or major vascular pathologies require rapid assessment of vascular integrity using imaging and immediate intervention to prevent rupture and life-threatening haemorrhage. Early haemodynamic stabilisation and timely surgical or endovascular management are critical to improve survival outcomes [14].

Abdominal and Gastrointestinal Emergencies

These include obstructive, perforative, and ischemic pathologies, where early clinical assessment, imaging confirmation, and timely operative decision-making are essential to prevent progression to sepsis or organ dysfunction.

Urological Emergencies

Obstructive and infectious urological conditions require prompt decompression, antimicrobial therapy where indicated, and supportive care to prevent renal compromise and systemic complications. Timely access to care pathways significantly influences outcomes, particularly in resource-constrained or disrupted healthcare settings [27].

Cross-Cutting Principles and Emerging Support Systems

Across all categories, adherence to structured protocols, timely surgical intervention, and coordinated perioperative care remain fundamental. Emerging digital health systems and simulation-based monitoring tools may support perioperative planning and risk assessment, although their integration into routine emergency practice is still evolving [28].

Pharmacological interventions in acute care

Pharmacological management in acute care can be structured according to major clinical domains to align therapeutic priorities with underlying pathophysiology. Pharmacological management continues to play a key role in stabilising life-threatening conditions, requiring appropriate drug selection, dosing, and timely administration [34,35].

Infectious Emergencies

Sepsis and severe infections require immediate initiation of broad-spectrum antimicrobial therapy, which is a critical determinant of survival [12]. Subsequent escalation or de-escalation based on clinical response and diagnostic findings is essential to balance efficacy with antimicrobial stewardship [26]. Coordinated care pathways further enhance outcomes when pharmacological treatment is integrated with supportive care [20].

Oncological and Metabolic Emergencies

Patients with malignancy-related complications may present with acute issues such as bowel obstruction, thromboembolic events, or metabolic derangements, necessitating rapid pharmacological intervention tailored to the underlying condition and altered physiology [5]. Careful risk assessment is required to ensure safe and effective drug administration.

Analgesia and Sedation

Pain and anxiety control are central to acute care management, improving physiological stability and facilitating diagnostic and therapeutic procedures. These interventions must be individualised to avoid adverse effects such as respiratory depression or haemodynamic instability [13,17].

Anticoagulation and Thromboembolic Management

Anticoagulant therapy is guided by the balance between thrombotic risk and bleeding potential, with decisions influenced by haemodynamic status and underlying pathology.

Emerging and Supportive Pharmacological Strategies

**Figure 1:**
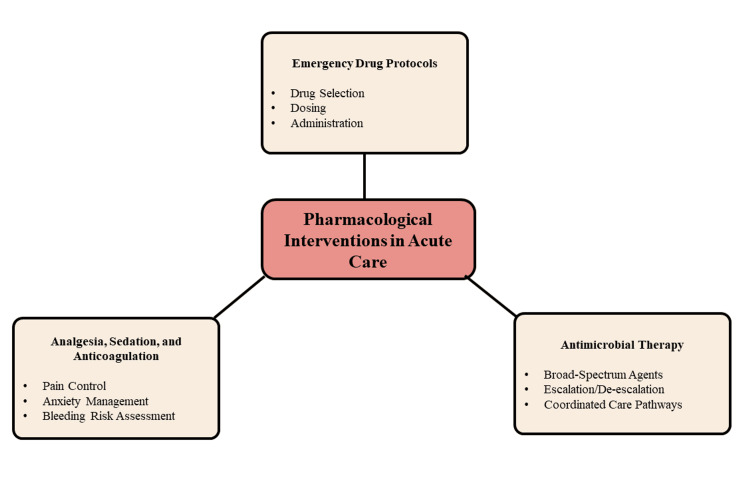
Pharmacological Interventions in Acute Care Created by authors using Microsoft PowerPoint

Advanced decision-support systems and machine learning-assisted tools are being explored to optimise drug selection, dosing, and risk prediction in critical care, although clinical oversight remains essential [27]. Personalised pharmacotherapy approaches continue to evolve, offering potential improvements in treatment precision and outcomes. Figure 1 illustrates the core components of pharmacological interventions in acute care management.

Pharmacological interventions in acute care

Pharmacological interventions in acute care can be categorised based on underlying clinical priorities to improve clarity in therapeutic decision-making. Pharmacological treatments should be initiated promptly, with careful drug selection and appropriate dosing to stabilise the patient and prevent progression to life-threatening conditions [36]. Emergency drug protocols must account for altered physiology, organ dysfunction, and unpredictable pharmacokinetics commonly seen in critically ill patients [6].

Infectious Emergencies

Infectious crises represent a major indication for urgent pharmacological intervention, where early administration of antimicrobial agents significantly influences clinical outcomes [21]. Empiric broad-spectrum therapy followed by pathogen-directed treatment based on diagnostic confirmation remains a cornerstone of sepsis management and improves survival. Zoonotic and systemic infections, including conditions such as brucellosis, require targeted combination therapy and timely adjustment based on clinical response [17].

Principles of Antimicrobial Optimisation

Therapeutic strategies emphasise early initiation, appropriate spectrum coverage, and subsequent refinement of therapy to balance treatment efficacy with antimicrobial stewardship. These approaches align with established sepsis management frameworks and highlight the importance of integrating pharmacological treatment with ongoing clinical and diagnostic evaluation.

Adjunct pharmacological management in acute care is primarily directed toward established, evidence-based interventions that support stabilisation and definitive treatment. Core components include analgesia, sedation, anticoagulation, and condition-specific pharmacotherapy tailored to the acute presentation. The management of analgesia and sedation requires careful titration to achieve symptom control while avoiding adverse effects such as respiratory depression or haemodynamic instability [13]. Adequate pain control improves physiological stability and facilitates diagnostic and therapeutic procedures. Anticoagulation strategies are applied in thromboembolic emergencies and must be individualised based on bleeding risk, haemodynamic status, and underlying pathology. In acute neurological emergencies, such as intracerebral haemorrhage, pharmacological management focuses on blood pressure control, reversal of coagulopathy, and prevention of secondary brain injury [6]. These interventions are supported by protocol-driven approaches in emergency care, with increasing use of clinical decision-support systems to optimise drug selection and dosing in high-acuity settings, although clinician oversight remains essential. Speculative or non-standard therapies, including plant-derived compounds and experimental nanotechnology-based agents, are not part of routine emergency pharmacological practice and are therefore not emphasised in this review. Pharmacological precision remains a key component of effective acute care management.

Interdisciplinary and critical care integration

Management of acute surgical and medical emergencies requires close collaboration between emergency medicine, surgical services, and intensive care units, forming a coordinated workflow from initial presentation to definitive care [37]. The emergency teams provide early identification, rapid stabilisation, and initiation of diagnostic and therapeutic pathways, whereas surgical teams deliver timely procedural or operative intervention when necessary. Intensive Care Units (ICUs) support advanced organ monitoring, haemodynamic optimisation, and continuous management of complex physiological derangements [38].

These interdisciplinary processes represent established workflow models in emergency care, particularly in high-acuity conditions such as sepsis and septic shock, where coordinated transition from emergency resuscitation to critical care enables timely antimicrobial therapy, source control, and organ support. Such integrated approaches are associated with improved clinical outcomes, including reduced mortality and organ dysfunction. Multidisciplinary decision-making models combine the expertise of emergency physicians, surgeons, intensivists, anaesthesiologists, and specialty consultants, facilitating balanced risk assessment, individualised treatment planning, and rapid adaptation to clinical deterioration [39,40].

In large-scale healthcare crises such as the COVID-19 pandemic, multidisciplinary coordination has been essential for managing increased numbers of critically ill patients, standardising treatment strategies, and optimising resource utilisation [41]. Collaboration between surgical and anaesthesia teams during intraoperative procedures also contributes to improved outcomes by optimising ventilation strategies and reducing postoperative pulmonary complications in high-risk emergency surgeries.

Timely escalation of care and structured post-intervention monitoring represent key evidence-supported components of integrated emergency management. Continuous physiological monitoring allows early detection of complications, treatment failure, or evolving organ dysfunction [22,14]. Cardiac emergencies such as acute valvular regurgitation and myocardial infarction require close postoperative and critical care monitoring to guide stabilisation and further intervention [4]. Structured surveillance systems also support early recognition of complications such as emergence delirium in vulnerable populations, enabling prompt supportive care and prevention of secondary injury [29]. Overall, while interdisciplinary integration reflects an ideal care model, its clinical value is supported by improved continuity of care, enhanced decision-making, and better patient outcomes in acute emergency settings. Table 3 shows the roles of interdisciplinary teams in integrated emergency and critical care management.

**Table 3:** Interdisciplinary and Critical Care Integration in Acute Emergency Management ICUs: Intensive Care Units

Care Component	Primary Discipline(s) Involved	Key Responsibilities	Clinical Impact	Reference
Early emergency stabilization	Emergency medicine	Rapid assessment, initial resuscitation, pathway initiation	Improves early survival and timely escalation	[18]
Definitive procedural care	Surgical services	Emergency operative and procedural interventions	Prevents disease progression and complications	[6]
Advanced organ support	Intensive care units	Hemodynamic optimisation, ventilatory and organ support	Reduces organ failure and mortality	[7]
Multidisciplinary decision-making	Emergency, surgery, ICU, anaesthesia	Integrated risk assessment and treatment planning	Enhances care coordination and adaptability	[4]
Post-intervention monitoring	Critical care and speciality teams	Continuous surveillance and complication detection	Supports early intervention and outcome optimisation	[3]

Emerging technologies and innovations in emergency management

The use of emerging technologies is transforming emergency management through improvements in diagnostic accuracy, therapeutic precision, and system coordination [42].

Current Standard and Widely Adopted Practices

Clinical decision support systems based on artificial intelligence are increasingly integrated into emergency workflows for risk stratification and early detection of deterioration, particularly in acute cardiovascular emergencies and critical care monitoring [43,44]. Tele-emergency medicine and remote triage systems are established in routine practice, enabling rapid specialist consultation in stroke evaluation, trauma triage, and sepsis management, especially in resource-limited or geographically remote settings [45-47]. Minimally invasive and endovascular interventions represent standard care in several acute conditions, including cardiogenic shock and acute vascular emergencies, where they provide rapid stabilisation with reduced procedural morbidity compared to open surgery [48,49].

Evolving Practices With Growing Clinical Adoption

AI-assisted analytics for medication optimisation and adverse event prediction are increasingly being incorporated into acute care workflows, although their use varies across institutions. Similarly, structured telemedicine platforms are expanding beyond triage to support ongoing monitoring and multidisciplinary coordination in emergency and critical care settings. Minimally invasive approaches in gastrointestinal emergencies are also evolving, with increasing use in selected high-risk patients, although outcome standardisation and long-term evidence continue to develop [50].

Future Directions and Exploratory Technologies

Advanced AI applications, including fully automated decision-making systems and predictive modelling of complex clinical trajectories, remain investigational and require further validation before widespread adoption. Emerging digital health technologies such as simulation-based monitoring systems and integrated predictive platforms are in early stages of implementation and are not yet standardised across emergency care systems. These distinctions highlight that while several technologies are already embedded in clinical practice, others represent evolving or future directions, with their clinical impact dependent on further evidence, validation, and integration into established care pathways.

Limitations and future directions

There are multiple limitations of narrative synthesis that apply to this review. The broad heterogeneity of acute surgical and medical emergencies, variations in study design, and differences in healthcare settings do not allow direct comparison across conditions and interventions. A substantial proportion of the available evidence is derived from observational studies, narrative reviews, and consensus guidelines rather than high-quality randomised controlled trials, particularly in time-sensitive emergency contexts. The rapidly evolving nature of technologies and treatment strategies also limits the durability of the conclusions. Publication bias and underreporting of negative outcomes may further influence interpretation, thereby affecting generalisability across diverse clinical settings. In addition, the broad scope of this review and the inclusion of heterogeneous evidence sources may reduce the strength and specificity of the clinical conclusions, as findings are synthesised across diverse conditions, populations, and healthcare environments rather than derived from uniform comparative data. As a result, conclusions should be interpreted as indicative of current practice trends and overarching principles rather than definitive, condition-specific recommendations.

High-quality prospective studies and pragmatic trials focusing on integrated emergency care pathways should be prioritised in future research. Standardised risk stratification tools applicable across mixed surgical and medical emergencies require further development and validation. Greater emphasis should be placed on real-world evaluation of artificial intelligence, tele-emergency systems, and minimally invasive interventions to establish safety and effectiveness. Strengthening interdisciplinary frameworks and improving equitable access to emergency services, particularly in resource-limited settings, remain essential. Dynamic clinical guidelines that incorporate evolving evidence and technology-based insights may further enhance responsiveness and improve outcomes in emergency care delivery.

## Conclusions

Acute surgical and medical emergencies remain among the most demanding challenges in contemporary healthcare due to their unpredictable presentation, rapid clinical deterioration, and high risk of adverse outcomes. This review highlights the importance of timely assessment, structured stabilisation, and evidence-informed management across diverse emergency conditions. Integrated diagnostic strategies, protocol-driven pharmacological interventions, and timely surgical or procedural care form the foundation of emergency response. The role of interdisciplinary collaboration among emergency medicine, surgical services, and intensive care units is an important contributor to continuity, safety, and outcome optimisation. Advances in point-of-care diagnostics, minimally invasive procedures, and supportive critical care practices have contributed to improvements in selected areas of emergency management, although their impact may vary across settings. Emerging technologies, including artificial intelligence-assisted decision support and tele-emergency platforms, represent evolving approaches with potential to enhance early recognition, triage accuracy, and clinical coordination, particularly in resource-constrained or high-demand settings. However, their widespread clinical effectiveness and integration remain dependent on further validation, standardisation, and clinician training. Persistent gaps in standardised risk stratification, variable guideline adherence, and unequal access to emergency services underscore the need for ongoing research and system-level innovation. Strengthening evidence generation, interdisciplinary frameworks, and adaptive care models may help support more resilient and patient-centred emergency systems capable of addressing evolving clinical and technological challenges.
